# Beyond UHC: Monitoring Health and Social Protection Coverage in the Context of Tuberculosis Care and Prevention

**DOI:** 10.1371/journal.pmed.1001693

**Published:** 2014-09-22

**Authors:** Knut Lönnroth, Philippe Glaziou, Diana Weil, Katherine Floyd, Mukund Uplekar, Mario Raviglione

**Affiliations:** Global TB Programme, World Health Organization, Geneva, Switzerland

## Abstract

Tuberculosis (TB) remains a major global public health problem. In all societies, the disease affects the poorest individuals the worst. A new post-2015 global TB strategy has been developed by WHO, which explicitly highlights the key role of universal health coverage (UHC) and social protection. One of the proposed targets is that “No TB affected families experience catastrophic costs due to TB.” High direct and indirect costs of care hamper access, increase the risk of poor TB treatment outcomes, exacerbate poverty, and contribute to sustaining TB transmission. UHC, conventionally defined as access to health care without risk of financial hardship due to out-of-pocket health care expenditures, is essential but not sufficient for effective and equitable TB care and prevention. Social protection interventions that prevent or mitigate other financial risks associated with TB, including income losses and non-medical expenditures such as on transport and food, are also important. We propose a framework for monitoring both health and social protection coverage, and their impact on TB epidemiology. We describe key indicators and review methodological considerations. We show that while monitoring of general health care access will be important to track the health system environment within which TB services are delivered, specific indicators on TB access, quality, and financial risk protection can also serve as equity-sensitive tracers for progress towards and achievement of overall access and social protection.

## Abstract 2

In a Collection Review for the Universal Health Coverage Collection, Knut Lönnroth and colleagues propose a framework for monitoring both health and social protection coverage, as well as their impact on TB epidemiology.

This paper is part of the PLOS Universal Health Coverage Collection.

**Summary Points**

1. The WHO has developed a post-2015 Global TB Strategy emphasizing that significant improvement to TB care and prevention will be impossible without the progressive realization of both universal health coverage and social protection. This paper discusses indicators and measurement approaches for both.
2. While access to high-quality TB diagnosis and treatment has improved dramatically in recent decades, there is still insufficient coverage, especially for correct diagnosis and treatment of multi-drug resistant TB.
3. Continued and expanded monitoring of effective coverage of TB diagnosis and treatment is needed, for which further improvements to existing surveillance systems are required.
4. Many households face severe financial hardship due to TB. Out-of-pocket costs for medical care, transport, and food are often high. However, income loss is the largest financial threat for TB-affected households.
5. Consequently, the financial risk protection target in the post-2015 Global TB Strategy—“No TB affected families experience catastrophic costs due to TB”—concerns all direct costs as well as income loss. This definition is more inclusive than the one conventionally used for “catastrophic health expenditure,” which concerns only direct medical costs.

## Universal Access and Social Protection in the Post-2015 Global TB Strategy

The WHO has developed a post-2015 global tuberculosis (TB) strategy (Box 1) with an overall goal to end the global TB epidemic, defined as a global TB incidence of <10/100,000, by 2035 [1]. The strategy acknowledges that poor health care access and inadequate financial risk protection are main hurdles, and stresses that it will be impossible to achieve full implementation of required interventions without the progressive realization of social protection and universal access to general health services. Tracking progress on universal access and social protection for those affected by TB is therefore a key part of the monitoring framework for the new strategy.

**Box 1. The Post-2015 Global Tuberculosis Strategy**

**VISION:** **A world free of tuberculosis**

- zero deaths, disease, and suffering due to tuberculosis

**GOAL:** End the global tuberculosis epidemic

**MILESTONES FOR 2025:**

- 75% reduction in tuberculosis deaths (compared with 2015);
- 50% reduction in tuberculosis incidence rate (fewer than 55 tuberculosis cases per 100,000 population)
- No affected families facing catastrophic costs due to tuberculosis

**TARGETS FOR 2035:**

- 95% reduction in tuberculosis deaths (compared with 2015)
- 90% reduction in tuberculosis incidence rate (fewer than 10 tuberculosis cases per 100,000 population)
- No affected families facing catastrophic costs due to tuberculosis

**PRINCIPLES:**

1. Government stewardship and accountability, with monitoring and evaluation
2. Strong coalition with civil society organizations and communities
3. Protection and promotion of human rights, ethics, and equity
4. Adaptation of the strategy and targets at country level, with global collaboration

**PILLARS AND COMPONENTS**

1. **INTEGRATED, PATIENT-CENTERED CARE AND PREVENTION**
  - Early diagnosis of tuberculosis including universal drug susceptibility testing; and systematic screening of contacts and high-risk groups
  - Treatment of all people with tuberculosis including drug-resistant tuberculosis; and patient support
  - Collaborative tuberculosis/HIV activities; and management of co-morbidities
  - Preventive treatment of persons at high-risk; and vaccination against tuberculosis
2. **BOLD POLICIES AND SUPPORTIVE SYSTEMS**
  - Political commitment with adequate resources for tuberculosis care and prevention
  - Engagement of communities, civil society organizations, and public and private care providers
  - Universal health coverage policy; and regulatory frameworks for case notification, vital registration, quality and rational use of medicines, and infection control
  - Social protection, poverty alleviation, and actions on other determinants of tuberculosis
3. **INTENSIFIED RESEARCH AND INNOVATION**
  - Discovery, development, and rapid uptake of new tools, interventions, and strategies
  - Research to optimize implementation and impact, and promote innovations

TB remains a major public health challenge worldwide, with an especially high burden among the poorest individuals in low- and middle-income countries, and among other marginalized populations. About 3 million of the estimated 8.6 million people who develop TB each year are either not diagnosed or are diagnosed but do not access TB care that meets international quality standards [2]. The TB coverage gap is proportionally larger for multidrug-resistant TB (MDR-TB) because of the low coverage of drug susceptibility testing, insufficient access to second-line TB drug regimens, and insufficient programmatic and health system capacity to deliver care [3]. More than two-thirds of the estimated 0.5 million annual incident MDR-TB cases are undetected [2].

The coverage gap is large despite much improved availability of quality-assured basic TB diagnosis and treatment services over the past two decades [4]. Owing to the considerable positive public health impact of effective TB care [5] and the high cost-effectiveness of TB diagnosis and treatment [6], the global standard has been set that all essential diagnostic tests for TB and all TB medicines should be government-funded and free of charge for patients. In 2013, 89% of countries reported to WHO that TB diagnosis (sputum smear microscopy) was provided free of charge within government-run services, while first-line TB medicines were free of charge in 87% of reporting countries [2]. However, within outside services unlinked to the national programmes, such as in the private sector, user charges are common. Domestic government financing dominates provision of free-of-charge TB services, while international support is still essential in many low- and lower-middle income countries [7].

Although, basic TB services are available, in principle, free of charge in almost all countries, the process required for people with TB to reach facilities that provide those services is often time-consuming, cumbersome, and costly [8]–[10]. In most countries, services for people with TB are fully integrated within general health services [11],[12]. Therefore, geographical and financial access barriers for general health services are access barriers for TB services as well. Before being diagnosed with TB, people often face large costs for the consultations and tests required for the differential diagnosis, symptomatic treatment, antibiotic trial treatments, and hospitalization. One consequence is that some people will wait too long before seeking care and, when they do, may not complete the procedures required for a definitive diagnosis. For those who do complete the diagnostic process, any pre-existing financial buffer may have been exhausted, which adds to the challenge of completing a treatment course that lasts a minimum of six months [9],[10].

The total cost of TB illness and care can be catastrophic, causing further impoverishment and forcing people to resort to potentially irreversible coping mechanisms, such as taking on large loans or selling property or livestock. The risk is particularly high for people who require lengthy treatment for MDR-TB and people in the lowest socioeconomic groups [13]. The financial burden may translate into augmented health risks: those who face catastrophic costs are more likely than others to interrupt treatment and have poor TB treatment outcomes [14],[15]. Those patients who delay care seeking or fall out of care owing to an inability to pay may further transmit disease with public health consequences. Moreover, further impoverishment because of TB has an impact on the household and community level, increasing future TB risks in already vulnerable groups. Poverty is associated with an increased risk of being infected with and developing active TB [16], delayed TB diagnosis [17], poor TB treatment adherence [14],[15],[18],[19], and higher TB case fatality [20].

For all of these reasons, universal health coverage (UHC)—defined as “universal access to needed health services without financial hardship in paying for them” [21]—is essential. To ensure good access to TB services, general health services need to be covered, not only TB-specific diagnosis and treatment [11]. However, paying out-of-pocket for health services is only a part of the financial burden. Non-medical costs and income losses often constitute a larger financial burden than the direct medical costs. A recent systematic review found that the total costs of TB for patients and affected families on average corresponded to more than half a yearly income [13]. Out-of-pocket medical expenses only accounted for an average of 20% of the total cost, while income losses accounted for 60%, and non-medical expenses for 20% [13]. Therefore, while measures towards minimization of out-of-pocket health care expenditures are essential for financial risk protection, they are not sufficient. Social protection interventions, designed to prevent or mitigate non-medical costs and income loss during the lengthy treatment [22],[23], are also required. The linkages between actions towards UHC and broader social protection are being increasingly addressed, especially when improving equity is a key aim [24].

There is growing evidence that social protection interventions can help improve, directly and indirectly, clinical outcomes for people with TB, especially among the poorest. Economic support in combination with other types of assistance, has been associated with improved uptake of TB services [25], improved adherence to treatment of latent TB infection [18],[25], and improved outcomes of treatment for drug-susceptible- [26],[27] and MDR-TB [28]–[30]. Such support is often included in TB grants from the Global Fund to Fight AIDS TB and Malaria [31]. In all societies, TB affects the poorest individuals the most, therefore interventions guided by research need to be tailored to ensure the maximum impact for this group [32]–[35]. This approach is also relevant for other health priorities, especially those in which the health condition is highly debilitating and recommended interventions require repeated or timely interaction with health services, such as for many non-communicable diseases.

## Evolving Post-2015 Monitoring Framework

The post-2015 Global TB Strategy includes two epidemiological targets: a 95% reduction in deaths caused by tuberculosis and a 90% reduction in the tuberculosis incidence rate between 2015 and 2035. These targets are much more ambitious than the current United Nations Millennium Development Goal (MDG) goal to “halt and begin to reverse TB incidence by 2015” and the MDG-related target to halve death rates between 1990 and 2015 [36]. The new strategy also has a financial risk protection target: 0% of TB-affected families facing catastrophic costs because of tuberculosis. The latter target is seen as a prerequisite for the two epidemiological targets, therefore, the projected year for reaching it is already 2020.

**Figure 1:**
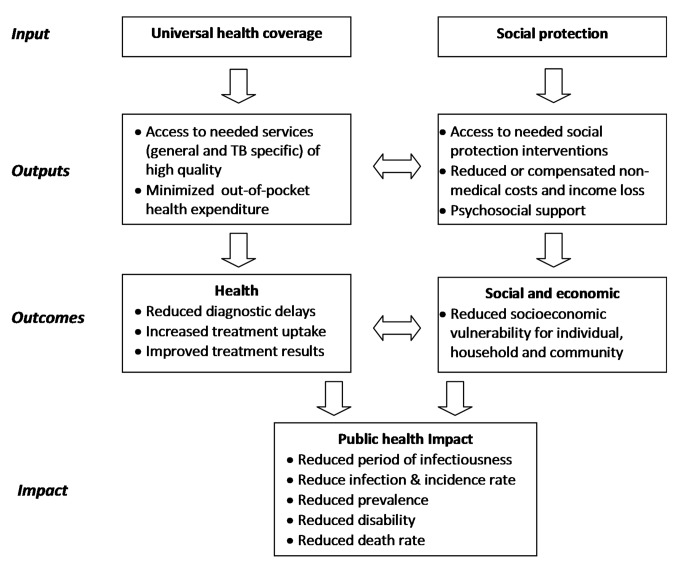
Framework to illustrate the interrelationship between universal health coverage, social protection, TB outcomes, and public health and social impact.

**Figure 2:**
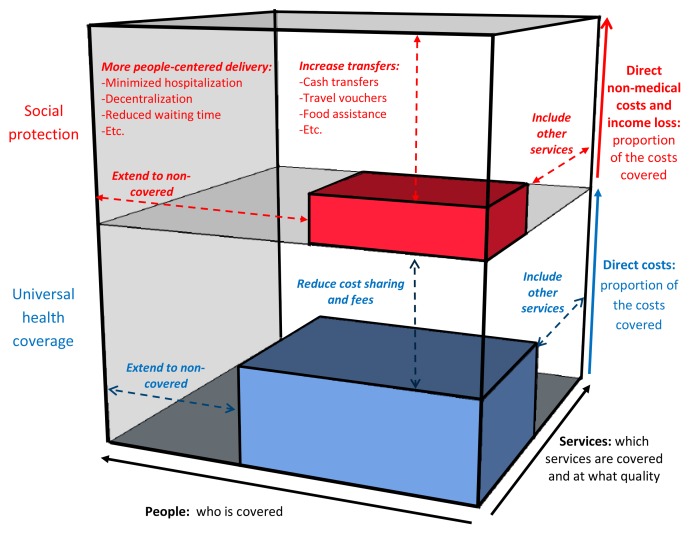
The three dimensions of universal health coverage, with the added dimension of financial risk protection against non-medical costs. Adapted from World Health Report 2010 [21]. Elements in red are non-medical costs and additional interventions within health care and beyond to provide financial protection.

A putative framework for the impact of UHC and social protection on TB epidemiology is provided in Figure 1. Figure 2, which builds on a conceptual UHC framework first presented in the World Health Report 2010 [21], shows schematically how social protection adds one level of improved financial risk protection beyond what can be achieved through UHC. Figure 2 also indicates specific areas of monitoring in the context of TB care and prevention.

Possible TB-specific indicators related to UHC and social protection that could be used as part of the post-2015 global TB strategy and a broader post-2015 development framework are listed in Table 1. They are grouped into input, output, outcome, and impact indicators, following the framework in Figure 1 and consistent with the proposed overall UHC monitoring framework proposed by Boerma and colleagues [37]. Indicators should be disaggregated by socioeconomic and demographic status to properly identify gaps and progress towards equitable access. While all listed indicators are of potential operational relevance for national or sub-national monitoring and performance improvement, to simplify efforts only some of them should be part of global monitoring.

**Table 1:** Indicative TB-related universal access and social protection indicators and targets.

Level	Indicator	Definition	Sources	Global Target	Interpretation
**Inputs**	Situation assessment of UHC financing policies and mechanisms done and how TB is addressed within these (Y/N)	Situation assessment includes; (a) population, service and cost coverage assessment; (b) payment mechanisms, conditions for reimbursement, quality standards, and accreditation of providers; (c) the extent to which TB diagnosis and treatment, and related TB care services are covered within revenue generation or insurance packages	NA	% of countries	Situation assessment is essential for planning of TB services and their link with general health services and general health insurance and other health financing schemes
	Situation assessment done on how TB is addressed within social protection (Y/N)	Situation assessment includes; (a) mapping of any schemes available to those affected by TB (e.g., sickness insurance, disability pension, cash transfer, food assistance, etc.); (b) the intended target groups for the schemes; and (c) how schemes are designed to prevent or mitigate adverse financial and social consequences of TB	NA	% of countries	Situation assessment is essential for planning of TB services, in particular TB patient support interventions, and their link with general social protection schemes
**Outputs**	Number of TB diagnostic facilities per population	*Numerator:* Number of TB diagnostic facilities in the country*Denominator:* Population	NTP management data	Country level only	Sufficient geographical availability of TB diagnostic facilities is essential for early TB diagnosis
	Number of TB treatment facilities per population	*Numerator:* Number of TB treatment facilities in the country*Denominator:* Population	NTP management data	Country level only	Sufficient geographical availability of TB treatment services is essential to ensure complete treatment initiation and adherence
	Proportion of bacteriologically confirmed TB cases among all newly diagnosed TB cases	*Numerator:* Number of people with suspected pulmonary TB tested using WHO recommended rapid diagnostics*Denominator:* All people investigated for pulmonary TB	NTP TB laboratory and treatment register	>90%	A high proportion indicates good diagnostic quality and less risk of false positive diagnosis based on clinical assessment only
	Percentage under-reporting of diagnosed cases of TB	*Numerator:* Number of cases diagnosed but not reported*Denominator:* Total number of diagnosed cases	Record linkageInventory study	<10% cases unreported	This indicator measures TB care coverage in terms of linkage with all relevant public and private health providers diagnosing and treating TB
**Outcomes**	Number of notified TB cases	Number of TB cases notified in a year	TB surveillance system	Country level only	Level and trend should be interpreted in relation to documented efforts to improve access and diagnosis, as well as other epidemiological parameters
	Ratio of notified cases over estimated incident TB cases in the same year^a^	*Numerator*: Notified TB cases in a year*Denominator:* Estimated number of incident TB cases in the same year	TB surveillance system.WHO TB incidence estimates	As close as possible to 100%	A high proportion can be achieved through a sufficient geographical coverage of TB diagnostic services; general UHC coverage; and availability of appropriate social protection sensitive to TB
	Percentage of persons diagnosed with bacteriologically confirmed TB who start TB treatment	*Numerator*: Number of persons diagnosed with bacteriologically positive TB*Denominator*: Number of persons diagnosed with bacteriologically positive TB registered for TB treatment	NTP TB laboratory and treatment registers	100%	A high proportion can be achieved through a combination of sufficient geographical coverage of TB treatment services; general UHC population, service and cost coverage; and availability of appropriate social protection sensitive to TB
	TB treatment success ratio in new TB cases	*Numerator*: Number of new TB cases started on treatment and successfully treated*Denominator*: Total number of new TB cases started on treatment	NTP TB treatment register	>90%	A high proportion can be achieved through a combination of sufficient geographical coverage of TB treatment services; general UHC population, service and cost coverage; and availability of appropriate social protection sensitive to TB
	Percentage of people with TB with some form of social or economic support benefits	*Numerator:* Number of people receiving care for TB and receiving social protection benefits*Denominator:* Total number of people receiving care for TB	NTP register cross-checked with other registers	Country level only	The higher proportion, the better coverage of social protection interventions. However, the appropriate level of coverage is context specific and depends on profile/needs of patients
	Percentage of people with TB who face catastrophic costs^b^	*Numerator:* Number of people receiving care for TB and experiencing catastrophic costs (direct medical, non-medical and income loss combined) due to TB illness and TB care*Denominator:* Total number of people receiving care for TB	Periodic surveys of patients receiving care for TB	0%	Low percent with catastrophic cost means that UHC mechanisms protect people from high direct medical costs and appropriate social protection prevent or mitigate high indirect costs
**Impact**	TB Incidence		Surveillance data. Vital statistics.	↓90% by 2035	Rate of decline associated with degree of effective UHC and social protection coverage
	TB prevalence		Surveys.	Country only	
	TB deaths		Modelling	↓95% by 2035	

All indicators should be disaggregated by sex and age.

a Should be disaggregated for drug-susceptible and drug-resistant TB.

b These indicators should be disaggregated by age, sex, and socioeconomic status, or in the case of geographical coverage mapped against poverty mapping.

NTP, national tuberculosis programme; NA, not applicable.

Most of these indicators are already well-established and captured in existing recording and reporting systems. Since the inception of “DOTS” (the WHO's global TB strategy 1995–2005) and expanded in the Stop TB Strategy, 2006–2015, there has been a strong focus on monitoring and evaluation [38],[39]. The Global Tuberculosis Report [2] has summarised key indicators for all Member States annually since 1997. This process was made possible by the broad adoption of standardised recording and reporting practices enabling assessment of diagnostic procedures, prescribed treatments, and patient-based cohort analysis [40]. Benchmarks for quality of care have been put in place; the most important being a TB treatment success rate of at least 85% among new TB cases initiating treatment. Coverage indicators include both the geographical coverage of diagnostic and treatment facilities and the “case detection ratio” (the ratio of TB cases registered for treatment in a given year over estimated incident TB cases for the same year).

Early mathematical modelling predicted that achieving at least a 70% case detection ratio and an 85% treatment success rate would lead to a significant decline in TB transmission and incidence [41],[42]. These two indicators and associated 70/85 targets were adopted by the World Health Assembly in 1991 [43] and also included in the MDG monitoring framework (Goal 6, indicator 24) [44],[45]. Together, they attempt to measure the effective coverage of TB services and have become central to the monitoring of programme performance [2]. The targets have become more ambitious in response to more ambitious epidemiological impact targets (Table 1). Additional output and outcome indicators for detailed monitoring of special areas of attention, such as collaborative TB/HIV activities, diagnosis, and management of MDR-TB [45], and contribution of different care providers to TB detection and treatment [46],[47], should be included on the basis of country context.

## Methodological Considerations for the Choice and Measurement of Indicators

### Service Quality Indicators

Standardized TB recording and reporting systems generate, when used correctly, solid direct measurements of the quality of TB diagnosis and treatment, through patient-based laboratory and treatment registers. Cohort analysis is routinely done for TB patients in almost all countries, which provides information about the proportion of patients who are successfully treated, and can be disaggregated by age and sex [2]. Geographical disaggregation is possible at the district and sometimes sub-district level, and can be mapped against geographical characteristics (e.g., urban/rural), poverty indices, and other development indicators. This analysis can provide some measures of equity in access to quality services; however, most standard information systems do not include socioeconomic data at the individual level, and therefore more precise assessments of equity require operational research.

### Coverage Indicators

TB service availability data, such as the number of diagnostic and treatment facilities per capita, can be obtained from national TB programme management data and through general service availability mapping. While these indicators can be easily measured in most settings, they only provide a partial indication of actual service access and coverage.

The most attractive coverage indicator is conceptually one that accurately measures the proportion of incident TB cases that are correctly diagnosed with TB and put on appropriate treatment. Combined with data on the proportion of TB patients who are successfully treated, such an indicator can provide information about effective coverage (i.e., the proportion of people who fall ill with TB who are diagnosed and successfully treated). However, accurately measuring the denominator for this indicator is challenging. Throughout the past decade, TB incidence has proved difficult to measure or estimate in most countries, and therefore estimates of the case detection ratio are often uncertain. Incidence surveys (very large population-based cohort studies) are resource-intensive and highly impractical. For this reason, no country has ever successfully implemented a nationwide representative TB incidence survey. When incidence is derived from routine case notifications, there is uncertainty about the number of cases not captured by routine surveillance. In high-income countries with high-performance TB surveillance and health systems, case notification systems capture almost all incident cases. In other countries, however, routine case notifications provide biased data because of under-diagnosis (cases not diagnosed) and under-reporting (cases diagnosed by health practitioners but not reported to public health authorities). WHO derives estimates of incidence in most low- and middle-income countries through a standard analytical framework that uses the available surveillance and programmatic data (including TB mortality data from vital registration systems). The outcome has considerable uncertainty and may be biased. Inventory studies to measure TB under-reporting are increasingly being used and will lead to better estimation of the total number of detected cases. When certain assumptions are met, capture-recapture modelling using data collected in inventory studies can also be used to estimate under-diagnosis and TB incidence [48],[49].

Models based on an assumed fixed relationship between the annual risk of TB infection (ARTI) and incidence of TB disease have been used in the past, but have been abandoned since it has been shown that the underlying assumptions are incorrect in most settings [50]. Predicting trends in TB incidence according to intervention scenarios has been attempted through models of TB transmission [51]. There is a considerable amount of literature on mathematical models of TB transmission, often with subtle differences in the structure but large differences in predictions [52].

In recent years, a growing number of national population-based surveys of TB prevalence have been conducted, to better measure the disease burden caused by TB and to help monitor progress towards the epidemiological impact targets [53]. These surveys can also provide direct (though cross-sectional) data on coverage of TB services and also provide invaluable information about access, health seeking behaviour, and health care utilization, disaggregated by socioeconomic status demographic factors. However, TB prevalence surveys are expensive and require large sample sizes and are only feasible in high TB burden settings [54].

The main solution for the future is to strengthen the performance of TB surveillance systems so that they cover all providers of health care and minimize the level of under-reporting. WHO has developed a checklist, the “standards and benchmarks for TB surveillance and vital registration systems,” to assess a national surveillance system's ability to accurately measure TB cases and deaths [55]. On the basis of the assessment, gaps and unmet monitoring and evaluation needs in national surveillance systems can be identified and strategies can be developed to address those needs.

### Financial Risk Protection Measurements

Mapping existing social protection schemes and assessing the extent to which people with TB are covered by them and actually use them is essential. Several political, financial, and operational challenges may limit real access for the intended target population. Monitoring coverage and outcomes will help identify the need for investigating and addressing bottlenecks. Such monitoring and related targets are relevant at the national level only, since the availability and types of schemes vary greatly among countries. Once schemes have been mapped out, countries can design specific data entry forms as part of the routine TB recording and reporting system (or general health information systems), to monitor coverage among people with TB. Whether such data collection is done routinely or as ad hoc research should be decided at the country level. In some settings, where social protection databases exist, there is the potential for establishing efficient cross-checking across databases.

Financial risk protection should be monitored globally and nationally to assess progress towards the catastrophic cost target for TB-affected households. Repeat patient surveys will be required unless a simple proxy indicator can be included in routine monitoring. Data can be collected at randomly selected facilities or in sentinel sites.

For overall (not TB specific) monitoring of financial risk protection, WHO has proposed to use “catastrophic health expenditure,” defined as the direct health care expenditures corresponding to >40% of annual discretionary income (income after basic needs, such as food and housing, are met) [21]. An alternative approach is to measure the incidence of impoverishment (the number of people pushed into poverty and/or further into poverty) due to out-of-pocket expenditures [37]. Neither of these indicators includes non-medical costs of care and income loss in the numerator.

The TB-specific indicator for financial risk protection includes all care-related expenditures, including non-medical direct costs, as well as income losses (Figure 2). Measuring income losses is normally more difficult than measuring direct medical costs [56], and will therefore require special attention. A different measurement approach and a different definition of “catastrophic” (compared to that used for general “catastrophic health expenditure”) will be needed. One option would be to adopt the definition of “total costs corresponding to >10% of annual household income,” which has been proposed by Ranson [57]. Another possibility is to use a cut-off of 20% of annual income, which has been associated with a doubling in the risk of a poor TB treatment outcome [15]. Finally, it may be possible to use generic or locally defined irreversible coping strategies as proxy indicators for catastrophic costs. WHO and partners have developed a tool-kit for the measurement of patient costs [58]. It provides options for measurement approaches and guides country adaptation of the generic survey instrument [58]. This tool-kit was primarily intended to be a “diagnostic” tool, which should help countries identify the main cost drivers and thereby inform policy decisions on how to reduce patient costs and related access barriers. It can also be used for monitoring of progress towards financial risk protection.

### TB as an Equity-Sensitive UHC Tracer

TB affects the most vulnerable individuals [16],[59]–[62], and eliminating their catastrophic costs is fully aligned with cross-agency recommendations to address equity in access [24]. TB indicators are therefore appropriate for inclusion in a broader UHC monitoring framework, as separate tracer indicators or as part of composite indicators. In Table 2, TB indicators are mapped against the preferred attributes of intervention coverage indicators for overall UHC, adapted from Boerma and colleagues [37].

**Table 2:** TB indicators mapped against the preferred attributes of intervention coverage indicators for general universal health coverage monitoring [49].

Preferred Attribute	Assessment for TB	Comment
Is a health priority based on burden of disease addressed by an intervention	Yes	TB is a leading cause of death and morbidity, especially among the poorest in the poorest countries
Is it a cost-effective intervention	Yes	TB diagnosis and treatment are among the most cost-effective public health interventions ever documented
Includes a measure of quality (sometimes referred to as “effective coverage”)	Yes	There are several robust quality indicators, including diagnostic quality, verified treatment results, and case fatality
Credible methods exist to identify the population needing the intervention, i.e., the denominator	Partly	This is the most challenging aspect of TB coverage monitoring since the true TB incidence is difficult to measure directly. However, in settings where UHC exists and under-reporting is minimal, TB notifications provide a good proxy of TB incidence. Population prevalence is directly measurable in the highest burden countries and the TB death rate is, in principle, measurable in all countries through improved vital registration.
Credible methods exist to identify the population receiving the intervention, i.e., the numerator	Yes	The information about number of people receiving quality-assured TB treatment is readily available in almost every country
Can be routinely measured: health management information systems or periodic household survey	Yes	There is an internationally recommended standard TB information system that is used in almost all countries
Equity disaggregation is possible by household wealth/income, gender, residence, and other key stratifiers	Yes	Disaggregation by age, sex, and geographical area is available from standard records. Additional disaggregations require research with special data collection
Measureable in comparable way across countries	Yes	TB case definition, diagnostic quality, treatment regimens, and treatment outcomes are internationally standardised

It has been proposed that UHC monitoring should have a particular focus on the poorest 40% of the population [37]. This monitoring would entail a focus on the vast majority of people with TB, globally and within most countries. Poor TB performance usually translates into ineffective coverage for the poorest 40%, while, conversely, very good overall TB performance in a country indicates that the poorest 40% are reached with at least one key health intervention. However, when gaps exist, as they still do in most settings, there is a need to assess equity in access through disaggregation by socioeconomic status. It is quite likely, though assessing equity requires further study, that it is the poorest 10% or 20% of individuals, rather than the poorest 40%, who are left out when TB performance is suboptimal. The same argument can be used for TB as a proxy to measure the performance of social protection for the poorest or most marginalized segments of society.

## Conclusions

The monitoring of TB service coverage and quality to inform and improve performance has been an essential element of WHO's global TB strategy for decades. A new post-2015 strategy that places strong emphasis on the importance of universal access and social protection for effective TB care and prevention has been developed. Refined coverage and quality indicators for TB prevention and care, as well as new indicators for financial risk protection have been developed by WHO. The coverage indicators also cover social protection interventions, while the financial risk protection indicator encompasses both the direct and indirect costs of TB. The monitoring framework therefore moves towards a vision of going beyond the conventional concept of UHC through the inclusion of social protection elements, either as an integral part of UHC, a “UHC+” concept, or as a separate package with robust links to UHC.

A few indicators should be used for global monitoring, such as the TB-affected success rate, the case detection ratio, and the proportion of TB-affected households experiencing catastrophic costs, as well as their impact on TB incidence and death rate. Additional operational indicators are needed for national and sub-national monitoring. Indicators on TB access, quality, and financial risk protection can serve as proxies for overall coverage and social protection progress.

Ensuring strong linkages to extend access to health and broader social protection is relevant not just for TB but for all health priorities, including chronic conditions, such as many non-communicable diseases that are debilitating, entail major indirect medical costs for frequent interaction with health services, and can lead to catastrophic income loss and other adverse socioeconomic consequences. Improving coordinated monitoring and evaluation of whether affected persons are receiving health services and social protection benefits is one way of stimulating improvement in access, and thereby better health and socioeconomic outcomes.

## Abbreviations

MDG: Millennium Development Goal
MDR: multidrug-resistant
TB: tuberculosis
UHC: universal health coverage
